# Behavioral Diversity Across Classic Rodent Models Is Sex-Dependent

**DOI:** 10.3389/fnbeh.2019.00045

**Published:** 2019-03-06

**Authors:** José Colom-Lapetina, Anna J. Li, Tatiana C. Pelegrina-Perez, Rebecca M. Shansky

**Affiliations:** Department of Psychology, Northeastern University, Boston, MA, United States

**Keywords:** sex differences, fear conditioning, forced swim, darting, coping

## Abstract

Symptoms of trauma and stressor related disorders such as post-traumatic stress disorder (PTSD) often develop well after the traumatic experience has occurred, and so identifying early predictors of risk or resilience is important for the implementation of interventional therapies. For example, passive coping strategies such as tonic immobility and peritraumatic dissociation during the trauma itself are risk factors for the developments of PTSD, especially in women. However, discrete, sex-specific coping responses that predict later outcomes in animal models have not been rigorously defined. Recently, we identified an active, escape-like response exhibited primarily by a subset of female rats in a classic auditory fear conditioning task (“darting”). Here, we asked whether darting during conditioning predicted active responding in a single forced swim (SFS) session to study the potential for darting to reflect a trait-like behavioral strategy that translated across stress models. Male and female Sprague-Dawley (SD) rats were tested in auditory fear conditioning acquisition and memory tests to identify Darters, and then a 15-min SFS 2 weeks later. We observed a significant effect of sex in conditioned freezing behavior, with males exhibiting greater freezing than females across conditioning and testing trials in comparison to females. However, females demonstrated higher velocities in response to shock presentations, and were more likely to exhibit darting behavior in response to the conditioned stimulus (CS). In SFS measures, females engaged in active behaviors such as climbing, head shaking, and diving in greater proportions than males, while males spent more time immobile throughout testing. Despite females exhibiting a more diverse behavioral repertoire in both tests, Darters did not differ from Non-darters in any SFS measure. These results suggest that the propensity to dart does not reflect a simple hyperactivity, and that despite conceptual overlap across the two tests (inescapable stress exposure and the ability to measure active vs. passive coping), the behavioral strategies engaged by an individual animal in each are likely driven by discrete mechanisms. We discuss potential challenges in interpretation of standard behavioral outcomes in classic models across the sexes, and consider the potential need for novel models that better tap into motivational states in females.

## Introduction

Selection of an appropriate response to threatening stimuli is a highly conserved evolutionary mechanism that can prove advantageous to the survival of a species. However, these responses can threaten survival when they become maladaptive (McEwen and Stellar, 1993; Commons et al., 2017). Post-traumatic stress disorder (PTSD) is characterized by heightened, unremitting fear responses following exposure to a traumatic event. However, not all individuals who are exposed to trauma will go on to develop PTSD (Yehuda and LeDoux, 2007). The fact that symptoms of PTSD do not present themselves until months after the trauma has passed means that careful attention should be devoted to the delineation of markers that may serve to identify populations that are at significant risk. While a great deal of work has been devoted to identifying biological risk factors (such as being female), little is known as to how these relate to the selection of threat responses that contribute to the development of PTSD (Becker et al., 2007; Dai et al., 2017). The observation that certain peri-traumatic risk factors such as tonic immobility or peritraumatic dissociation correlate strongly with subsequent development of PTSD suggest that more efficient therapeutic interventions may arise from the study of how specific behavioral manifestations during trauma may be indicative of susceptibility or resilience to stress-induced pathology (Kassam-Adams et al., 2012; Thomas et al., 2012; Möller et al., 2017).

PTSD is characterized by intrusive memories and frequent re-experiencing of the traumatic episode (Clohessy and Ehlers, 1999). Pavlovian models of fear conditioning allow researchers to probe the neural basis of fear learning and memory by assessing behavioral responses to cues that have been associated with aversive outcomes (Schafe et al., 2001). Patients with PTSD exhibit potentiated acquisition of conditioned fear, as well as deficits in extinction learning (Milad et al., 2009; VanElzakker et al., 2014). Therefore, Pavlovian fear conditioning is a useful tool to study key neural circuitry that may go awry in patients suffering from PTSD (Phelps and LeDoux, 2005; Sijbrandij et al., 2013). In rodents, the amount of time spent freezing during the presentation of a conditioned stimulus (CS) is interpreted as an index of the strength of the association created, as well as the intensity of the fear itself (Fanselow, 1980). However, these models operate under the assumption that freezing is the only way in which fear can be expressed and quantified. Recent experiments from our lab characterized a novel, escape-like, active fear response known as “darting” that occurs primarily in a subpopulation of females during CS tone presentations (Gruene et al., 2015). Animals that darted during fear conditioning exhibited enhanced extinction retention, suggesting that engaging in a more diverse behavioral repertoire during aversive learning can promote cognitive flexibility in the future. In another study, we similarly found that in a forced swim test (FST), females are more likely to engage active coping strategies (Colom-Lapetina et al., 2017). Altogether these findings suggest that there are sex-specific, adaptive responses to threatening stimuli that are the result of individual variability within subsets of populations, and that these differences may help to identify potential markers of resilience. However, whether active responding during fear conditioning reflects a broad propensity towards active coping that translates across the two models has not been tested. The purpose of the current work, therefore, was to investigate the possibility that darting represents a sex-specific predictor of active coping in other forms of inescapable stress.

## Materials and Methods

### Subjects

Young adult (~8–9 weeks at time of arrival) male (*n* = 23) and females (*n* = 22) Sprague-Dawley (SD) rats weighing 275–300 g and 225–250 g, respectively were housed in pairs at the Nightingale Animal facility at Northeastern University. Subjects were kept on a 12:12 light:dark cycle with access to food and water *ad libitum*. All procedures were conducted in accordance with the National Institutes of Health guide for the Care and Use of Laboratory Animals and approved by the Northeastern University Institutional Animal Care and Use Committee. Experimenters were male and female.

### Behavioral Testing

#### Apparatus and Stimuli

Seven to 10 days after arrival at the vivarium, rats underwent fear conditioning trials as described in Gruene et al. (2015) in one of four identical chambers. Chambers are made with aluminum and Plexiglass walls (Rat Test Cage, Coulbourn Instruments, Allentown, PA, USA). A shock generator (Model H13–15; Coulbourn Instruments) was attached to the metal stainless steel rod flooring and the chambers were lit with a small house light. Each chamber was enclosed in sound-isolation cubicle (Model H10–24A; Coulbourn Instruments) and an infrared digital camera in each cage recorded behavioral testing from above. Once each trial was completed, chamber grid floors, trays, and walls were thoroughly cleaned with ethanol in preparation for the next session.

#### Fear Conditioning and Memory Test

On day 1, rats were allowed to explore cage for 4 min before five tone (CS) presentations (habituation), followed by seven conditioning trials [CS-unconditioned stimulus (US) pairings] on day 1. The CS was a 30 s, 5 kHz, 80 dB SPL sine wvave tone, which co-terminated with a 0.5 s, 0.7 mA footshock (US) during conditioning trials. On day 2, rats were placed back in fear conditioning apparatus and exposed to two CS-only presentations to assess successful acquisition of conditioned fear. Time freezing and velocity traces during both trials were recorded using Ethovision software (Noldus) and analyzed using custom Python code^1^.

#### Single Forced Swim Session

All procedures were conducted during the animal’s light cycle, under standard lighting. Animals were returned to facilities immediately following Fear conditioning paradigm and allowed to reacclimate to facilities for 14 days prior to single forced swim (SFS). At approximately 1100, rats were transferred from facility and placed in different room from testing and animal facilities for 1 h before forced swim. Subjects were placed individually in a plexiglass cylinder measuring 50 cm high and 20 cm wide that was filled with clean tap water (25 ± 2°C) to a height of 32 cm. Animals were subjected to a 15-min swim period. Once the session ended, animals were removed from the tank and carefully dried for 30 min under a lamp in home cages before being returned to animal facilities. We tested four animals per day, counterbalanced for sex. Forced swim sessions were recorded with a built-in camera on an iMac computer and Noldus Ethovision XT software was used to score total time of immobility. Ethovision detection settings were calibrated to match those of experimenter scores. Subjects were considered immobile when floating and displaying minimal limb movements necessary to keep heads above water. Head shaking, climbing, and diving behavior were quantified *via* video hand-scoring by experimenters, blind to the animal’s experimental group. Head shaking was described as short, vigorous bouts of head shaking and climbing was considered when rats approached walls of cylinders and displayed vertical limb movement (Colom-Lapetina et al., 2017).

### Statistical Analysis

Behavioral data were analyzed using two-way ANOVAs with corrected *post hoc* tests for multi-trial or block behavioral tests (Fear conditioning, Forced Swim). Paired *t*-tests were used for single comparison tests (time spent diving), and chi-square tests to assess sex differences in subpopulations of Darters and Divers.

## Results

### Fear Conditioning Behavior

**Figure 1:**
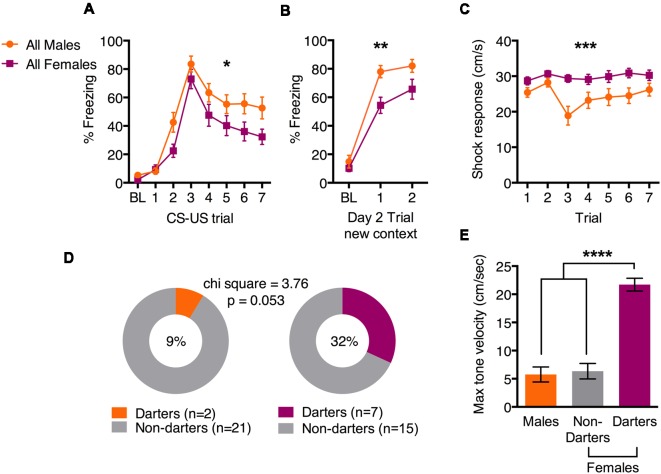
Sex differences in active and passive behavior during fear conditioning. **(A)** Females froze less than males during fear conditioning tone presentations. **(B)** Females froze less than males during a tone test in a new context 24 h after fear conditioning. **(C)** Females exhibited greater shock responses than males during fear conditioning, as measured by the maximum velocity reached. **(D)** Proportions of male and female cohorts that exhibited darting behavior. **(E)** Maximum tone velocity differed between female Darters and both males and female Non-Darters. *p < 0.05 main effect of sex; **p < 0.01 main effect of sex; ***p < 0.001 main effect of sex; ****p < 0.0001 adjusted *post hoc*, Darters vs. both males and female Non-darters.

Fear conditioning data are shown in Figure 1A. Freezing at baseline (BL) was low in both sexes, but freezing increased as tone-shock pairs progressed (main effect of trial *F*_(7,301)_ = 51.6; *p* < 0.0001), suggesting that both males and females learned the tone-shock association (Figure 1A). We also observed a main effect of sex (*F*_(1,43)_ = 4.3; *p* = 0.04), suggesting that overall, females froze less than males across trials. The next day, animals were presented with a two-tone test in a new context to evaluate consolidation. Again, both sexes increased freezing to the tone compared to BL (Figure 1B; main effect of trial (*F*_(2,86)_ = 113.5; *p* < 0.0001), and males froze more than females (main effect of sex *F*_(1,43)_ = 10.1; *p* = 0.003). We also evaluated the shock response across fear conditioning trials, and found that females responded with a greater velocity than males did (Figure 1C; main effect of sex: *F*_(1,44)_ = 14.6; *p* = 0.0004), consistent with our previous findings (Gruene et al., 2015). We then characterized males and females as “Darters” or “Non-darters” based on criteria set in Gruene et al. (2015). As we previously observed, a greater proportion of females qualified as Darters compared to males (Figure 1D; 32% vs. 9%, respectively. Chi square = 3.76; *p* = 0.053). We attribute the near-significance of this analysis to a potential slight underpowering of cohorts, but note that with larger cohorts, we have previously observed statistically significant sex differences in Darter populations (Gruene et al., 2015). To confirm that Darters and Non-Darters can be differentiated by behavior during the CS, we evaluated the maximum velocity reached by each animal during CS 3–7 (Figure 1E). A one-way ANOVA revealed a significant effect of group (*F*_(2,42)_ = 26.1; *p* < 0.0001) and *post hoc* tests revealed that Darters were significantly different from both Males and Non-darters (both comparisons *p* < 0.0001). Darters and Non-Darters did not differ in pre-shock locomotor activity (276.9 cm vs. 277.6 cm, respectively; *p* = 0.99, data not shown). Together, these data demonstrate that females exhibit slightly less conditioned freezing than males, but also exhibit more exaggerated shock responses and are more likely to engage in active conditioned responding, supporting our previous findings (Gruene et al., 2015).

### Forced Swim Behavior

**Figure 2:**
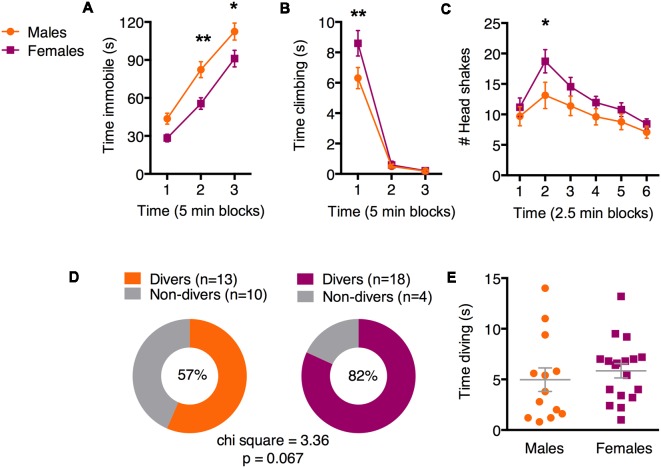
Sex differences in active and passive behavior during the forced swim test (FST). **(A)** Females exhibited less immobility than males in the last two single forced swim (SFS) time bins. **(B)** Females spent more time climbing than males during the first 5 min of the SFS. **(C)** Females exhibited more head shaking than males during early SFS time points. **(D)** Proportions of male and female cohorts that exhibited diving behavior. **(E)** Among Divers, males and females did not differ in time spent diving. *p < 0.05 adjusted *post hoc* males vs. females; **p < 0.01 adjusted *post hoc* males vs. females.

Two weeks after fear conditioning, all animals were exposed to a 15-min single SFS. We first evaluated immobility across three 5-min blocks of the SFS using a two-way ANOVA with factors of time and sex (Figure 2A). In both sexes, immobility increased with time [main effect of time (*F*_(2,86)_ = 172.6; *p* < 0.0001)]. However, females exhibited less immobility than males [main effect of sex (*F*_(1,43)_ = 10.53; *p* = 0.002)]. Adjusted Bonferroni *post hoc* tests revealed significant sex differences during time blocks 2 (*p* = 0.002) and 3 (*p* = 0.02), but not 1 (*p* = 0.14). Analysis of climbing behavior (Figure 2B) revealed a significant sex × time interaction (*F*_(2,126)_ = 3.98; *p* = 0.02). Adjusted Bonferroni *post hoc* tests revealed significant sex differences during time block 1 (*p* = 0.002), but not 2 (*p* = 0.99) or 3 (*p* = 0.99), suggesting that animals of both sexes exhibit climbing primarily during the first 5 min, but females engage in more climbing than males. We next evaluated head shakes in 2.5 min blocks (Figure 2C), because our video scorers noticed a reliable increase in head shaking during the second half of the first 5 min block, followed by a decrease over the remaining 10 min. We found significant main effects of time (*F*_(5,215)_ = 10.52; *p* < 0.0001) and sex (*F*_(1,43)_ = 4.15; *p* = 0.047). Adjusted Bonferroni *post hoc* tests revealed significant sex differences during time block 2 only (*p* = 0.04). Finally, we examined diving behavior. Like darting, diving is a discrete behavior that is exhibited by a subset of animals and may signify an alternate coping strategy. As with darting, we observed diving in a greater proportion of females compared to males (Figure 2D; 82% vs. 57%, respectively). Chi-square analysis revealed a near-significant sex difference (chi-sq = 3.4, *p* = 0.067). As above, we attribute the near significance of this analysis to slightly under-powering our *n*’s. Evaluation of time spent diving among animals that did engage in diving (“Divers”) suggested that while there was variability in both males and females, there were no overall sex differences (Figure 2E; unpaired *t*-test: *t* = 0.68, *df* = 29; *p* = 0.5). Together, these results suggest that in the SFS, females are more likely to engage in a more diverse set of behavioral strategies than males, who are more likely to engage in immobility.

**Figure 3:**
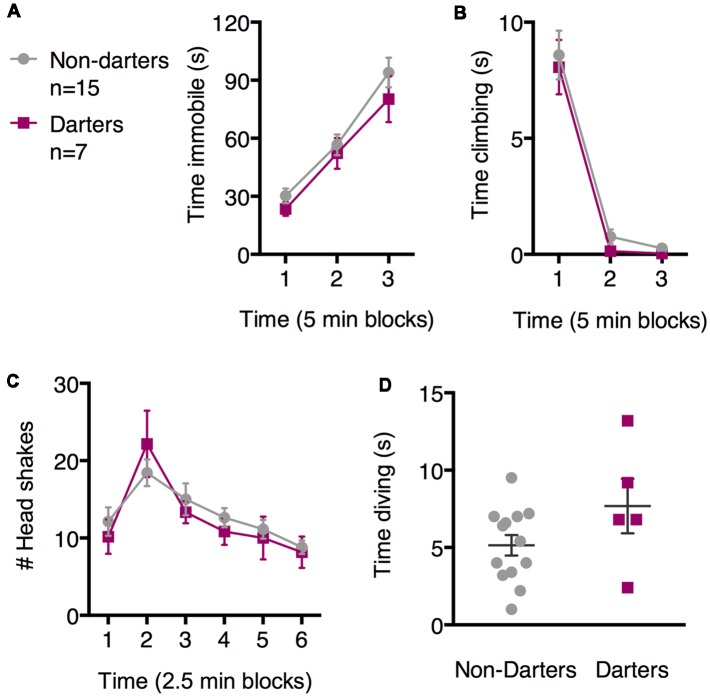
Darter and Non-darter females did not differ in the SFS. No statistically significant differences were observed between Darters and Non-darters in measures of immobility **(A)**, climbing **(B)**, headshakes **(C)**, or time spent diving **(D)**.

To determine whether these diverse SFS behaviors in females are related to darting during fear conditioning—in other words, whether behavioral diversity is a “trait” that can be observed across models—we next evaluated SFS behavior in females who were categorized as “Darters” or “Non-Darters” based on the occurrence of darting during fear conditioning. Seven females qualified as Darters. Darting during fear conditioning did not predict time spent immobile, climbing, or head shaking [Figures 3A–C; no main effects of darting on immobility (*F*_(1,21)_ = 0.93, *p* = 0.35), climbing (*F*_(1,20)_ = 0.38; *p* = 0.54), or head shaking (*F*_(1,20)_ = 0.12; *p* = 0.73]. Of the four females who did not engage in diving, two each were Darters and Non-Darters. A comparison of time spent diving between Darters and Non-Darters was not significant (Figure 3D; *t* = 1.68, *df* = 16; *p* = 0.11). However, we note that our “Diving Darter” *n* is fairly low (*n* = 5), and that this difference may have reached significance with more statistical power. In that case, diving and darting may represent common strategies across fear conditioning and SFS.

## Discussion

Our goals for the present study were to examine the relationships between individual behavioral strategies across classic behavioral paradigms. For decades, auditory fear conditioning and the FST have been used in laboratories across the world to study how the brain processes and responds to aversive experiences. However, the vast majority of studies using these models have been conducted in male rodents, and whether the standard behavioral metrics associated with each (e.g., freezing or immobility) sufficiently map onto the same motivational or emotional states in females has not been rigorously investigated (Shansky, 2015). Because stress-related disorders like PTSD are more prevalent in women (Breslau, 2009), it is imperative that we assess the validity our behavioral models in both sexes. A closer, more nuanced look at the range of behaviors an animal can exhibit in each may provide better insight into the core aspects of these diseases in both men and women.

Along these lines, we have recently found that females, but not males, will exhibit an active conditioned response during auditory fear conditioning (“darting”). This finding was important for several reasons. First, it demonstrates that measures of freezing alone are inadequate for assessing learning in classic Pavlovian models. Second, darting during conditioning was associated with enhanced extinction retention, suggesting that darting may reflect an alternate behavioral strategy that predicts long-term adaptive outcomes. Because we only observe darting in about 30%–40% of females, Darters may represent a discrete subpopulation of animals that are generally prone to active responding in stressful situations. The work here therefore represents our first attempt to identify additional behavioral patterns that can be predicted by darting. To test this idea, we identified Darters based on behavior during fear conditioning, and asked whether they were more likely to exhibit active responses during the SFS.

As we observed previously, more females than males exhibited darting during fear conditioning in the current study. Females also exhibited less conditioned freezing but greater shock responsivity than males, again supporting our previous findings. Importantly, the shock response data suggest that lower freezing in females is not due to lower perceived pain. In the SFS, we also observed more diverse behavioral patterns in females, who were more likely to exhibit climbing, head-shaking, and diving compared to males. These data are consistent with our previous findings (Colom-Lapetina et al., 2017), but do not map onto all studies of sex differences in the 2-day FST. For example, Kokras et al. (2017) have found that males are more likely to exhibit head shaking than females, and Rincón-Cortés and Grace (2017) recently reported more immobility in females compared to males. A comprehensive review of sex differences in the FST (Kokras et al., 2015) shows that there is no clear consensus as to the directionality of how males and females differ in immobility, swimming, and climbing. Important considerations that may contribute to these discrepancies include animal strain, vivarium light/dark cycle, water temperature, duration of the test, and disparities in scoring protocols across laboratories. Our primary goal here was not to examine sex differences in each test, but to identify behavioral measures that tracked across both tests, based on an animal’s propensity to dart during fear conditioning. However, we found that SFS measures were not different between females that qualified as Darters vs. Non-darters. To our knowledge, this is the first examination of non-freezing conditioned responses as a potential predictor of alternate coping strategies.

Other groups have attempted to categorize individual differences among cohorts in order to identify adaptive vs. maladaptive behavioral profiles and predictors. For example, a recent study divided male rats into “active” or “passive” coping groups based on behavior after a chronic social defeat experience (Grafe et al., 2018). In a subsequent FST, passive responders exhibited more immobility and less time swimming compared to non-defeated animals, which were statistically comparable to active responders. Unfortunately, female animals were not examined in this study. In another report, assessment of struggling behavior when briefly restrained on their backs (Back Test) allowed animals to be classified into “passive,” “active,” or “variable” coping groups. In males, these groups differed in behaviorally in the FST as well as in several physiological measures of stress, such as fecal corticosterone levels and cardiovascular activation (Hawley et al., 2010). However, although females could also be separated by passive, active, and variable coping, their classification was not predictive of behavioral differences in measures of diving in the FST, or of rearing and grooming in the Dry Land Maze task (Kent et al., 2017).

Our data suggest that in both auditory fear conditioning and the SFS, female SD rats exhibit greater behavioral diversity than males, engaging in a broader repertoire of coping behaviors. We emphasize the strain here because we and others have found that sex differences in the SFS can vary by strain (Kokras et al., 2015; Colom-Lapetina et al., 2017), and we have not yet assessed darting in other commonly used strains. The lack of differences between Darters and Non-darters in the SFS suggests that behavioral diversity itself is not necessarily an individual trait that transfers from one model to the next. In addition, this finding argues against the putative interpretation that Darters are simply hyperactive—a conclusion also supported by our findings here and previous that Darters do not exhibit greater BL locomotor activity (Gruene et al., 2015). Instead, we believe that Darters represent a true subpopulation of animals that switch strategies from passive (freezing) to active (darting) in conditioned fear paradigms. We look forward to dissecting the neural circuits and mechanisms that distinguish Darters from Non-Darters, as well as investigating the biological basis for darting’s prevalence in females.

One common pattern between our current findings and those of Kent et al. (2017) is that female behavior in the SFS could not be predicted by previous responding in a stress test. In contrast, similar approaches were successful in identifying consistent subpopulations in males, as described above. This discrepancy points to potential sex differences in the interpretive value of standard behavioral measures. As we have argued previously (Shansky and Woolley, 2016; Shansky, 2018), many common behavioral tests were developed and validated in male animals, and therefore the readouts we use to assess the same states in females may need to be adjusted. For example, a Principal Component Analysis of the elevated plus maze, which is traditionally used to measure anxiety, found that time spent in open vs. closed arms was most directly related to anxiety in males, but locomotor activity in females (Fernandes et al., 1999). Therefore, in models of stress coping and depressive-like behavior such as the FST, it is critical to determine which behaviors and metrics most accurately capture the motivational state of the subjects. Identifying sex differences in these methodologies could improve the translational value of these tests (Kokras and Dalla, 2017).

It is also important to recognize that different ethological demands in males and females may render active vs. passive responding differentially advantageous for each sex. For example, females may be more likely to survive a threat if they are able to escape it, while males may be better served by conserving energy and adopting more passive strategies. As we observed previously (Colom-Lapetina et al., 2017), a 2-day FST produces a sex-specific “learned helplessness” effect, such that immobility increased from day 1 to day 2 in males, but not females. One might interpret that to mean that females did not learn that escape from the FST is impossible, but an alternate interpretation could be that it is generally advantageous for females to try to escape stressful situations, and therefore they will maintain active coping responses longer than males will. This interpretation is supported by classic studies in the stress literature, showing that inescapable shock exposure impairs shuttlebox escape and active behaviors in the holeboard and elevated plus maze tests in males, but not females (Steenbergen et al., 1989, 1991). Given these early findings, it is perhaps unsurprising that we also observe a female-leaning propensity towards escape-like behaviors in standard fear conditioning and forced swim paradigms. This trend therefore warrants caution in interpreting effects such as those observed in the current study to mean that females are more adaptive or resilient than males simply because they exhibit greater behavioral diversity. In clinical populations, women are more susceptible to stress-related pathologies like PTSD (Breslau, 2009). If the ultimate goal of preclinical research is to improve disease treatment and prevention, we must accept the challenge of modifying our behavioral models and metrics to more precisely capture sex-specific vulnerabilities.

## Data Availability

The datasets generated for this study are available on request to the corresponding author.

## Author Contributions

JC-L and RS designed the experiments. JC-L and TP-P conducted all behavior testing. AL wrote the Python code for darting analysis. JC-L and RS analyzed the data and wrote the manuscript.

## Conflict of Interest Statement

The authors declare that the research was conducted in the absence of any commercial or financial relationships that could be construed as a potential conflict of interest.

## Footnotes

^1^https://github.com/annajxli/scaredy-rat
